# Water vapor thermal therapy of lower urinary tract symptoms due to benign prostatic obstruction: efficacy and safety analysis of a real-world cohort of 211 patients

**DOI:** 10.1007/s00345-023-04395-y

**Published:** 2023-05-04

**Authors:** Kathrin Bausch, Leutrim Zahiti, Michel Schrutt, Christian Wetterauer, Florian Samuel Halbeisen, Jan Ebbing, Hans-Helge Seifert

**Affiliations:** 1 Department of Urology, University Hospital Basel, Spitalstrasse 21, 4031 Basel, Switzerland; 2 University of Basel, Basel, Switzerland; 3 Surgical Outcome Research Center, University Hospital Basel, Basel, Switzerland

**Keywords:** Benign prostatic hyperplasia, Endoscopy, Lower urinary tract symptoms, Minimally invasive treatment, Prostate, Rezum, Thermal therapy, Water vapor therapy

## Abstract

**Purpose:**

This study assessed the efficacy, safety and durability outcomes of water vapor thermal therapy with Rezum in a real-world cohort of patients with lower urinary tract symptoms due to benign prostate obstruction.

**Methods:**

Consecutive, unselected patients undergoing Rezum treatment between January 2014 and August 2022 were candidates for this pragmatic, observational, longitudinal, single-center cohort study. Pre- and perioperative data were descriptively summarized. The primary outcome was surgical efficacy, determined by International Prostate Symptom Score (IPSS), Quality of Life (QoL) Score, maximum urinary flow rate (Qmax), post-void residual (PVR) volume and prostate volume (PV) at baseline, 2 months, 6 months, 1 year, 2 years, and > 2 years.

**Results:**

A total of 211 patients were enrolled for analysis. Overall, catheter removal was successful in 92.4% of patients after a median of 5 days. A preoperative catheter and the presence of a median lobe increased the risk of unsuccessful catheter removal. In total, 5.7% of patients were reoperated after a median of 407 days. Comparing baseline to the longest median follow-up, the postoperative IPSS decreased significantly by 65.7%, the QoL Score declined by 66.7% (both until a maximum median of 4.5 years) and Qmax improved by 66.7% (until 3.9 years). Post-void residual volume and PV were reduced by 85.7% (3.7 years) and 47% (4.0 years), respectively. Clavien–Dindo complication ≤ II occurred in 11.8%.

**Conclusion:**

Rezum is a safe minimally invasive treatment option in a real-world patient cohort with a beneficial improvement of micturition symptoms and voiding function during follow-up.

## Introduction

Transurethral resection of the prostate (TURP) is considered the gold standard among various procedures for surgical treatment of lower urinary tract symptoms (LUTS) caused by benign prostatic obstruction (BPO). However, due to changes in demography, surgical therapies are facing an increase in comorbidities leading to challenging surgical and anesthesiological decision making [1, 2]. Consequently, not only among an elderly and a potentially comorbid group of patients but also among the average patient cohort, the demand for minimal-invasive treatment (MIT) modalities is increasing. Water vapor thermal therapy (Rezum) by transurethrally injecting 103 °C water steam into the prostate is one of these MIT options and has demonstrated a beneficial efficacy and safety profile for the treatment of LUTS caused by BPO [3]. Previously published studies have either reported on relatively homogeneous collectives of patients [3–8] or focused on single specific baseline characteristics such as prostate volume (PV) or preoperative catheterization [9, 10]. To what extent these results can be translated to a real-world cohort—including preoperatively catheterized patients or patients with a large prostate or median lobe—has rarely been investigated yet.

We report short- to long-term efficacy and safety outcomes of Rezum therapy for BPO related to LUTS using an unselected real-world cohort.

## Materials and methods

### Study design and setting

We conducted a pragmatic, observational, longitudinal cohort study at the University Hospital of Basel, a tertiary care center. The Rezum system (Boston Scientific, Massachusetts, USA) has been introduced at the Department of Urology in January 2014. All Rezum procedures during the study period were performed according to the technique recently published [5, 11].

### Patient selection

All consecutive, largely unselected patients undergoing Rezum for BPO at our institution between January 2014 and August 2022 were eligible for study inclusion. Exclusion criteria were: (i) missing outcome data, (ii) refusal to participate in any clinical study project involving secondary use of routine health care data, (iii) suspicion of prostate cancer due to elevated PSA or digital-rectal exam unless prostate cancer was ruled out and (iv) patients with penile or sphincter prothesis.

### Data collection and definitions

Preoperatively, patient characteristics, International Prostate Symptom Score (IPSS), Quality of Life (QoL) Score, PV measured by transabdominal or transrectal ultrasound, maximum urinary flow rate (Qmax), post-void residual (PVR) volume, catheterization and medication were analyzed. Neither anticoagulants nor platelet aggregation inhibitors were paused for the intervention. Perioperative parameters included operative time, number and localization of water steam injections, duration of hospitalization and catheterization, intraoperative complications, and 30-day postoperative complications according to the Clavien–Dindo classification [12].

Standard catheterization time was 5 days unless indicated otherwise.

First routine follow-up visits were planned 1–13 months after the Rezum procedure. Additional follow-up visits were performed according to the evaluation of the attending physician (usually every 6–12 months). Follow-up was defined from the date of Rezum until the last consultation, up to October 2022, death or lost to follow-up. In case a second operative intervention was performed to treat BPO, follow-up was continued until the date of this intervention.

### Primary outcome

Primary outcome was operative efficacy determined by IPSS/QoL, Qmax, PVR and change in PV. Safety was assessed by intraoperative and 30-day postoperative complications according to the Clavien-Dindo classification [12].

### Statistical analysis

Relevant baseline and follow-up data were reviewed, with follow-up periods at 2 months, 6 months, 1 year, 2 years, and > 2 years after surgery.

All data were reported with appropriate descriptive statistics: non-normally distributed data was reported as median with interquartile range (IQR). We assessed the influence of a preoperative catheter, PV ≥ 80 ml and the presence of a median lobe on unsuccessful catheter removal and the need of reoperation using multivariable logistic regression.

Statistical significance was considered with *p* < 0.05. Statistical analysis was performed in R (R Core Team, http://www.R-project.org/).

## Results

### Patient characteristics

A total of 211 patients were included in the study. Median age was 68.0 years (IQR 61.0–77.0). Preoperative catheterization, PV ≥ 80 ml and a median lobe were present in 19.9%, 19.9% and 38.4% of patients, respectively (Table 1A).

**Table 1:** (A) Patient characteristics, (B) perioperative and postoperative efficacy outcomes, (C) potential risk factor analysis by multivariate logistic regression and (D) safety outcomes of Rezum

Characteristic	Rezum patients (*N* = 211)
(A) Patient characteristics	
Age in years, median (IQR)	68.0 (61.0–77.0)
ASA Score, median (IQR)	2 (2–3)
Preoperative medication for LUTS, *N* (%)	
None	71 (33.6)
Alpha blocker	94 (44.5)
5-alpha reductase inhibitor	4 (1.9)
Alpha blocker and 5-alpha reductase inhibitor	26 (12.3)
Alpha-blocker and anticholinergics	3 (1.4)
Alpha-blocker and beta mimetics	2 (0.9)
Alpha-blocker, anticholinergics, and beta mimetics	2 (0.9)
Anticholinergics, beta mimetics only or other combination	9 (4.3)
Anticoagulants/Platelet aggregation inhibitors, *N* (%)	
None	128 (60.7)
Acetylsalicylic acid	45 (21.3)
Clopidogrel	2 (0.9)
Acetylsalicylic acid and clopidogrel	5 (2.4)
Therapeutic heparin	1 (0.5)
Coumarins	8 (3.8)
Direct factor Xa-inhibitor	22 (10.4)
Preoperatic prostate characteristics preoperative IPSS, median (IQR)	18.0 (13.0–23.0)
Preoperative QoL Score, median (IQR)	3.0 (3.0–4.0)
Preoperative Q_max_ in ml/s, median (IQR)	8.4 (6.0–12.0)
Preoperative PVR in ml, median (IQR)	70.0 (30.0–180.0)
Preoperative prostate volume, median (IQR)	50.0 (36.0–70.5)
Indwelling catheter preoperative, *N* (%)	42 (19.9)
Prostate ≥ 80 ml, *N* (%)	42 (19.9)
Median lobe, *N* (%)	81 (38.4)

Characteristics	Rezum patients (*N* = 211)
(B) perioperative and postoperative efficacy outcomes	
Duration of operation in min, median (IQR)	10.0 (7.0–16.0)
Anesthesiologic procedure, *N* (%)	
General anesthesia	61 (28.9)
Analgosedation	118 (55.9)
Spinal anesthesia	7 (3.3)
Local anesthesia	25 (11.8)
Number of injections, median (IQR)	5.0 (3.0–7.0)
Number of injections median lobe in 81 patients with median lobe, median (IQR)	1.0 (1.0–2.0)
Intraoperative complications, *N* (%)	
None	195 (92.4)
Bleeding requiring coagulation for vision	6 (2.8)
Catheter for irrigation	10 (4.7)
Duration of hospitalization in days, median (IQR)	2.0 (2.0–3.0)
Number of treatments on an outpatient basis, *N* (%)	47 (22.3)
Total number of successful catheter removal, *N* (%)	195 (92.4)
Total days until successful catheter removal, median (IQR)	5.0 (5.0–7.0)
1st catheter removal	
Successful, *N* (%)	168 (79.6)
Days until successful 1st removal, median (IQR)	5.0 (4.0–6.0)
Not successful, due to elevated PVR, *N* (%)	20 (9.5)
Not successful, due to urinary retention, *N* (%)	23 (10.9)
Kept on catheter after 1st unsuccessful trial, *N* (%)	5 (11.6)
2nd catheter removal	38 (88.4)
Successful, *N* (%)	27 (71.1)
Days until successful 2nd removal, median (IQR)	22.0 (10.0–37.0)
Not successful, due to elevated PVR, N (%)	7 (18.4)
Not successful, due to urinary retention, N (%)	4 (10.5)
Successful catheter removal in 169 patients without a preoperative catheter, *N* (%)	168 (99.4)
Days until successful catheter removal, median (IQR)	5.0 (4.0–6.0)
Successful catheter removal in 42 patients with a preoperative catheter, *N* (%)	27 (65.8)
Days until successful catheter removal, median (IQR)	16.0 (5.0–36.0)
Successful catheter removal in 42 patients with prostate volume ≥ 80 ml, *N* (%)	34 (80.9)
Days until successful catheter removal, median (IQR)	6.0 (5.0–11.5)
Successful catheter removal in 81 patients with median lobe, *N* (%)	68 (83.9)
Days until successful catheter removal, median (IQR)	5.0 (5.0–7.0)
Total rate of reoperated patients, *N* (%)	12 (5.7)
Total days until reoperation, median (IQR)	407.0 (232.8–869.8)
Reoperation in 42 patients with a preoperative catheter, *N* (%)	1 (2.4)
Reoperation in 195 patients with successful catheter removal, *N* (%)	11 (5.6)
Reoperation in 16 patients without successful catheter removal, *N* (%)	1 (6.3)
Reoperation in 42 patients with prostate volume ≥ 80 ml, *N* (%)	4 (9.5)
Days until reoperation, median (IQR)	265.0 (126.0–407.0)
Reoperation in 81 patients with median lobe, *N* (%)	3 (3.7)
Days until reoperation, median (IQR)	714.0 (273.0–885.0)

Characteristics		
Parameters/potential risk factor	Unsuccessful catheter removal OR (CI), *p* value	Reoperation OR (CI), *p* value
(C) potential risk factor analysis by multivariate logistic regression		
Preoperative catheter	84.5 (14.7–1620), < 0.001	0.246 (0.0128–1.43), 0.198
Prostate volume ≥ 80 ml	2.35 (0.563–10.1), 0.238	4.29 (1.15–15.1), 0.024
Median lobe	9.06 (2.22–48.5), 0.004	0.464 (0.0973–1.67), 0.274

Characteristics	
Clavien-Dindo grade	Rezum patients (*N* = 211) *N* (%)
(D) safety outcomes of Rezum	
I	14 (6.6)
II	11 (5.2)
IIIa	0
IIIb	0
IVa	0
IVb	0
V	0

*CI* confidence interval, *N* number, *OR* odds ratio

### Perioperative data

39.3% of patients were operated under anticoagulants or platelet aggregation inhibitors. Operation time was 10 min (IQR 7–16). In two-thirds of the cohort, Rezum could be performed without general anesthesia. Patients received a median of 5 injections (IQR 3–7) and were hospitalized for a median of 2 days IQR 2–3) (Table 1B). 30-day postoperative complications are displayed in Table 1D.

### Catheter management and reoperations

In a total of 195 (92.4%) patients, the catheter could be successfully removed after a median of 5 days (IQR 5–7). Catheter removal was successful in 168 (99%) patients who had no catheter preoperatively after a median of 5 (IQR 4–6) days. In patients with a preoperative catheter, PV ≥ 80 ml and median lobe, catheters could be successfully removed in 65.8%, 80.9% and 83.9%, respectively (Table 1B). In total, 5.7% of patients were reoperated on a median of 407 days.

A preoperative catheter and the presence of a median lobe increased the risk of unsuccessful catheter removal, and patient with PV ≥ 80 ml had a higher risk of a reoperation (Table 1C).

### Functional outcome

**Fig. 1:**
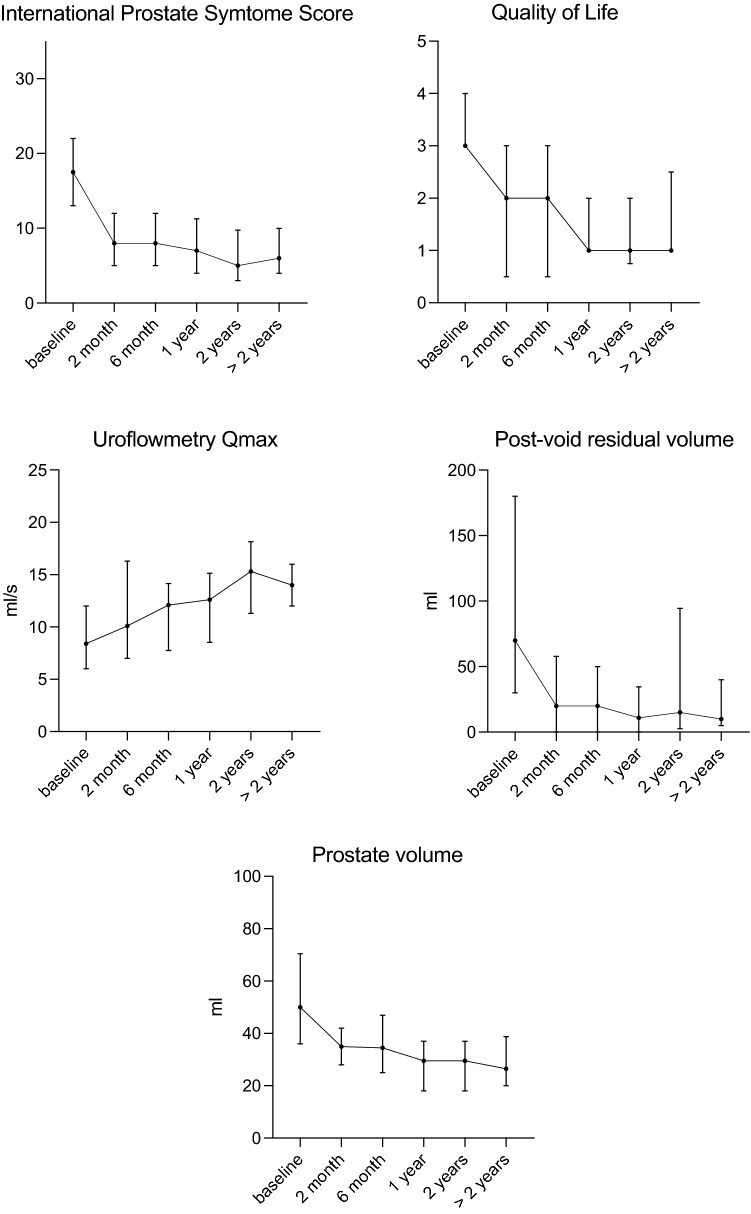
Short-, mid- and long-term micturition symptoms and voiding function (medians, IQR)

Comparing baseline to the longest median follow-up, the postoperative IPSS decreased significantly by 65.7%, the QoL Score declined by 66.7% (both until a maximum median of 4.5 years) and Qmax improved by 66.7% (until 3.9 years). Post-void residual volume and PV were reduced by 85.7% (3.7 years) and 47% (4.0 years), respectively (Fig. 1).

## Discussion

Our study results demonstrate a considerable, durable efficacy and safety profile of Rezum water vapor therapy of BPO-related LUTS in a real-world cohort. Our data show a considerable improvement in micturition symptoms with a reduction in IPSS by 54.3% and QoL by 33% already 2 months after treatment. Patients experienced an improvement in IPSS and QoL, which was maintained throughout the follow-up period of 4.5 years, confirming data from prior studies [3–8, 13]. Furthermore, voiding function improved after Rezum: an increase in Qmax by 20.2% was observed only 2 months after treatment, reaching maximum improvement with a 66.7% increase at 4 years after Rezum. Improvement in Qmax has been reported to be at 50–100% in other studies [5–8]. Prostate volume decreased by around 30% after no more than 2 postoperative months, which was maintained throughout the observational period. These results are in line with previously published cohorts [5–9, 13] and magnetic resonance imaging data showing a size reduction of about one-third [14]. Post-void residual volume also decreased early by 57% and was maintained through the observational period.

In the pivotal study of McVary et al. [3], rigid inclusion criteria concerning age (> 50 years), IPSS (≥ 13), Qmax (≤ 15 ml/s) and PV (30–80 ml) were applied, and other research groups had similar inclusion criteria for their patients [6–8]. We present comparable results on micturition symptoms and voiding function, though patients in our series were more heterogeneous with regard to their baseline characteristics. Compared to other real-world data, our study includes more patients over a longer follow-up period [13].

In previously published studies, the duration of postoperative catheterization was between 0 and 7 days [3–9]. In our series, catheterization time was 5 days in patients without preoperative catheter-dependent urinary retention. Trial void evaluations reported rates of postoperative acute urinary retention between 4.4 and 11.8% [3–9, 13]. In our cohort, the success rate of first catheter removal was 99% in patients without preoperative catheter-depend urinary retention after a median of 5 days. The catheter-free rate in the subgroup of patients with a preoperative catheter was 66% after a median of 16 days. A recently published study showed a slightly higher rate of 70.3% but after a longer period of time (26 days) [10]. These data suggest that time to first removal should potentially be prolonged in preoperatively catheterized patients. Furthermore, preoperative catheterization significantly increased the risk of an unsuccessful catheter removal potentially indicating the influence of additional causes of voiding dysfunction other than or additionally to BPO.

McVary et al. reported a surgical retreatment rate of 4.4% after 5 years with no statistical difference from the previous 4-year data [4]. In our real-world cohort, the reoperation rate seems to be slightly higher with 5.7% after a median time of 407 days. Interestingly, McVary et al. also reported that the majority of patients were reoperated in the first 2 years. Our cohort included a significant number of preoperatively catheterized patients or patients with a PV ≥ 80 ml or a median lobe. PV ≥ 80 ml seems to increase the risk of reoperation. These results show a conflicting picture compared to recently published data demonstrating a similar efficacy and safety profile independent of PV [9]. On the other hand, patients that had a higher PV also were frequently catheterized preoperatively.

Compared to other MIT methods, such as prostatic urethral lift (PUL) and prostate artery embolization (PAE), Rezum seems to outperform with regard to reoperation rates. As published by Miller et al. [15], reoperation rates are 6% per year after PUL. Following PAE, 21% of patients underwent TURP due to unsatisfying outcomes after 2 years [16]. We see a possible explanation in the lower reoperation rates in patients treated by Rezum compared to PUL and PAE, associated with the ablative nature of Rezum and the possibility of median lobe treatment.

For Aquablation—another MIT in used to treat LUTS associated with BPO—rates of reoperation after 2 years are similar to Rezum [17]. However, complication rates seem to be higher using Aquablation, most likely due to the ablative effect.

30-day complications in Rezum are low, especially no bleeding complications occurred even though one-third of the patients were under anticoagulation or platelet aggregation inhibitors. So far, only one of the previous studies stated to not pause anticoagulation when medically indicated [13] while we continued this medication in all patients. Consequently, we assume that Rezum is a safe procedure for patients under anticoagulation and platelet aggregation inhibitors.

This study is not without limitations. The pragmatic, observational design adds selection bias that may underestimate positive as well as negative outcomes. In addition, there is variability in the duration of follow-up. Some of the patients so far have incomplete data. We assume that patients with the worse outcome are more likely to have perceived more frequent consultations, suggesting a better outcome in the actual patient population. Though indication for Rezum was often an important factor for selecting Rezum as a treatment method in sexually active men, sexual outcome parameters such as ejaculation or erectile dysfunctions were rarely reported and for this reason not presented in this study.

## Conclusion

Rezum is an MIT option in a real-world cohort of patients with LUTS secondary to BPO. Micturition symptoms and voiding function improve stably over time. Preoperative catheterization and the presence of a median lobe increase the risk of unsuccessful catheter removal. Further studies are needed to determine and maybe extend patient groups for Rezum and to define the role of Rezum in the range of other BPO treatment options currently available.

## Data Availability

Raw data were generated at the Department of Urology, University Hospital Basel, Basel, Switzerland. Derived data supporting the findings of this study are available on request.
